# Editorial: Coronavirus Disease (COVID-19) and Its Psychobehavioral Consequences

**DOI:** 10.3389/fpsyg.2021.723282

**Published:** 2021-07-12

**Authors:** Severi Luoto, Marjorie L. Prokosch, Marco Antonio Correa Varella, Indrikis Krams, Corey L. Fincher

**Affiliations:** ^1^School of Population Health, University of Auckland, Auckland, New Zealand; ^2^Florida Institute of Built Environment Resilience, University of Florida, Gainesville, FL, United States; ^3^Department of Experimental Psychology, Institute of Psychology, University of São Paulo, São Paulo, Brazil; ^4^Institute of Ecology and Earth Sciences, University of Tartu, Tartu, Estonia; ^5^Department of Zoology and Animal Ecology, Faculty of Biology, University of Latvia, Riga, Latvia; ^6^Department of Biotechnology, Daugavpils University, Daugavpils, Latvia; ^7^Department of Psychology, University of Warwick, Coventry, United Kingdom

**Keywords:** COVID-19, evolution, pandemic, behavioral immune system, cognition, population health, evolutionary psychology

The presence of pathogens has imposed constant threats to human survival and reproduction. Selective pressures exerted by pathogens have shaped our array of immune functions—including physiological, psychological, and behavioral immune systems. Pathogens and epidemics have plagued humankind and our ancestors from their dawn, yet despite advances in hygiene and medicine, these threats remain with us today. In 2020–2021, pathogens have become a particularly salient part of everyday life as we have faced a worldwide outbreak of SARS-CoV-2.

We launched this Research Topic with the specific recognition that evolutionary approaches, which acknowledge the biological forces shaping and underlying human cognition and behavior, are uniquely positioned within psychology and behavioral science to offer insights on the responses to and outcomes of the COVID-19 pandemic. The collection of 14 articles published in this Research Topic has surpassed our original vision, introducing diverse evolutionary perspectives on various aspects of the COVID-19 pandemic. Varella et al. captured the importance of an evolutionary approach to COVID-19 by stating that “Everything in pandemics is stamp collection except in the light of evolution.” Research focused on pandemics without an explicit evolutionary framework can also be very valuable as it can offer the pieces of the jigsaw puzzle that evolutionarily oriented researchers need to integrate in their quest to understand the bigger picture.

## Game-Theoretical Approaches to the COVID-19 Pandemic

Cooperation and compliance with pandemic safety regulations are important facets of a public health response to limiting the spread of a virus such as SARS-CoV-2. These aspects of a public response to a pandemic can be fruitfully analyzed via evolutionary game theory. According to this approach, public health is a *public goods game*, and its maintenance depends on the contributions of a critical number of individuals, as discussed by Yong and Choy. The authors noted that this leaves room for defectors who can pursue their own interests without contributing to the common good of public health during the pandemic. Such free-riders can enjoy the benefits of decreased health risk from others' compliance with health policies despite failing to contribute to—or even undermining—public health themselves. Their non-compliance behavior may, when detected, also be punished for example through legal enforcement and penalties, as discussed in detail by Yong and Choy.

## Sex Differences in Pandemic Leadership

This scenario constitutes a public goods dilemma where various game-theoretic strategies may bear different payoffs according to the strategies of other “players” in the game, including the kinds of top-down policies that are implemented to curb the spread of the virus and to punish defectors. Luoto and Varella's review article on pandemic leadership established that female-led countries were better at minimizing COVID-19-related deaths in 2020. This outcome likely arose because of female leaders' stronger empathy, higher pathogen disgust, health concern, care-taking orientation, and dislike for the suffering of other people, as suggested by existing research on psychobehavioral sex differences in other domains (reviewed in detail in Luoto and Varella). Societal leadership and top-down policies comprise important factors that can shape human behavior so that the collective good of public health is prioritized over selfish individual actions, effectively overriding our evolved psychological mechanisms for selfish behavior so that we can reach a higher-level societal goal. As shown in Luoto and Varella's review article, male and female leaders, on average, placed slightly different emphases on different outcomes during a pandemic. Thus, men and women can have somewhat different “strategies” as players in the public goods “game” of the pandemic, in line with their evolved psychobehavioral dispositions.

## Prosocial vs. Selfish Behaviors in a Pandemic Context

Dinić and Bodroža demonstrated the crucial role of empathy and the importance of prioritizing higher-level societal goals during the COVID-19 pandemic. They studied the effect of six prosocial personality tendencies, selfishness as an antisocial personality trait, fear related to the pandemic, and empathy toward most vulnerable society members as determinants of protective behaviors against COVID-19 (e.g., washing hands, wearing a mask, and physical distancing). Altruism had a positive effect and selfishness had a negative effect on protective behaviors. Thus, an antisocial and selfish strategy can decrease the chances of personal survival and the survival of group members. Increased fear related to the pandemic had an important effect on people's protective behaviors. However, fear had no significant moderator effect in the relationship between personality traits and protective behavior. At the same time, the authors found that empathy acted as a mediator, helping to effectively promote health-responsible behaviors.

Other articles in the Research Topic have analyzed the characteristics of those more likely to act selfishly in such a “public goods dilemma” as the COVID-19 pandemic. Corpuz et al. reported that individuals with fast life history strategies had lower self-reported adherence to pandemic precautions, lower willingness to donate plasma, and lower endorsement of mandatory COVID-19 vaccination. This is consistent with the general idea of fast life history strategies being associated with bioenergetic investment in reproduction over longevity and health. Varella et al. combined this approach with their *eveningness epidemiological liability hypothesis*, which posits that evolved propensities for nocturnal activities—which manifest in contemporary life in activities such as restaurant dining and going to bars and nightclubs—might have become a dangerous liability against epidemiological control partly because SARS-CoV-2 persists in aerosols much longer during the night and indoors than outdoors during the day. Thus, life history strategies and chronotype, together with sex, can explain some of the individual differences in non-compliance with pandemic safety measures, although Varella et al.'s *eveningness epidemiological liability hypothesis* still requires direct empirical testing.

Norton et al. reported that during the early stages of the pandemic, Australians matched their behavior to perceived social norms, which were used to infer the seriousness of COVID-19. More specifically, there was a positive association between people's perceptions of others' adherence to social distancing recommendations, their perceptions of the seriousness of COVID-19, and their own adherence to social distancing recommendations. This finding has interesting implications for game-theoretical scenarios related to the pandemic, suggesting the existence of a “tit-for-tat” strategy. Self-reported adherence to safety measures was also positively linked to anticipated shame and to perceptions of the moral wrongness of non-adherence, indicating that the threat of social punishment may in part motivate adherence to pandemic safety guidelines—a kind of culturally sanctioned behavioral immune system.

## Behavioral Immune System

This kind of culturally enforced behavioral immune system is an important part of the collective fight against the pandemic because at the individual level, the SARS-CoV-2 virus can evade many of our evolved contagious disease avoidance tendencies. After all, even asymptomatic carriers of the virus are contagious, and most infected individuals have only very mild or no symptoms at all (Varella et al.). The absence of disease cues leads to an attenuated or even absent activation of contagious disease avoidance mechanisms, which can contribute to non-compliance with the pandemic safety measures, as noted by Varella et al. Ultimately, this highlights the need for top-down measures from health authorities and political leaders to eliminate the virus.

Comparisons of pre-pandemic behavioral measures with those acquired during the pandemic nevertheless showed heightened activation of individual-level behavioral immune system across three studies in this Research Topic. Studying Croatian populations, Hromatko et al. reported that preferred interpersonal distances, pathogen disgust, and germ aversion were higher during the pandemic than before. Conservatives and women were more likely to agree that the government should severely punish those who did not adhere to COVID-19 preventive measures (conversely, in the US, participants who reported being more conservative were less likely to endorse precautions surrounding COVID-19, as reported by Corpuz et al.). Furthermore, Croatian islanders had higher preferred distances with strangers and showed higher negative emotions toward foreigners than mainlanders did, suggesting higher behavioral immune system activation in islanders. This may arise from their more isolated geographical location, which makes them more vulnerable if a new pathogen finds its way into the population because isolated populations are typically shielded from exposure to various pathogens. Hromatko et al. concluded that when cues of risk of infection are high, xenophobic attitudes might serve as a steering wheel that keeps one from coming into close contact with possible disease carriers.

Models of disgust as functionally important for disease avoidance argue for its flexibility as disease circumstances change. Few tests of this idea exist. Stevenson et al. used the occurrence of the COVID-19 pandemic and data sets from prior studies to test this idea. They showed that people during the pandemic—a time when infection transmission is notably increased—reported heightened disgust sensitivity and germ aversion (a related construct) compared to similar samples from just a few years ago. They also showed that across the samples, while sex differences existed, the differences were consistent. Furthermore, they found that impulsivity was rather consistent across the samples. The consistency in sex differences and the similarity in impulsivity bolster the authors' conclusion that the increase in disgust sensitivity is related to the pandemic rather than reflecting nuances of the samples.

Miłkowska et al. also examined disgust fluctuations pre- and post-pandemic, examining two demographically similar cohorts of Polish women. Results partially supported the hypothesis that disgust increased during the early pandemic (April–May, 2020). Women from the pandemic cohort rated photographs of infection sources as more disgusting, scored higher in contamination sensitivity, and reported lower moral disgust than the pre-pandemic cohort. Cohorts did not differ in pathogen disgust. However, the pandemic cohort women reported higher state anxiety, which was positively associated with photograph disgust, contamination sensitivity, and pathogen disgust. As Hromatko et al. noted, the behavioral immune system therefore is a contextually sensitive pathogen detection and avoidance system which partially underlies social cognitions and patterns of interpersonal approach/avoidance motivations.

This view is further supported by the finding that the COVID-19 threat did not strengthen the relationship between disgust and homonegativity. Szymkow et al. reported a positive correlation between sexual disgust and negative attitudes toward gay men and lesbians in a Polish sample. However, pathogen disgust did not predict homonegativity, nor did homonegativity increase during the pandemic. This suggests that the behavioral immune system is not hypersensitive to homosexuality in the COVID-19 context. In a *sexual* context, in contrast, homonegativity was associated with increased disgust—possibly because of the association between sexually transmitted diseases and homosexuals, as discussed in Szymkow et al.

Despite the results showing increased behavioral immune system activation in the COVID-19 context, Gassen et al. reported that while participants with higher clinical risk for severe COVID-19 (calculated using weighted measures of demographic characteristics such as age, BMI, and sex, and pre-existing conditions such as cardiovascular disease or cancer) acknowledged their greater likelihood of experiencing severe illness if infected, they actually reported lower perceived likelihood of becoming infected. While such unrealistic optimism might improve the short-term psychological well-being of those at high risk, it can also lead to a level of carelessness that unnecessarily increases the risk of infection and severe COVID-19 for such high-risk individuals. As Gassen et al. noted, while optimism bias has evolutionary origins, it does not mean that unrealistic optimism is an “optimal” strategy in every situation, particularly when individuals experience a novel source (or scale) of risk that was not present in the environments under which optimism biases may have evolved. This perspective highlights the utility of the evolutionary mismatch hypothesis in the COVID-19 context, as discussed in detail by Varella et al.

## Reproductive Decision-Making, Postnatal Depression, and Eating Behaviors in a Pandemic Context

The mismatch perspective becomes even more relevant when considering large-scale existential threats like climate change. Gordon examined the relationships between mortality threats and reproductive decision-making using a life history theory framework. Extrinsic mortality threats (external threats to individual survival) are linked to greater reproductive effort, while existential threats (external threats to species survival) are relatively novel and remain unexamined in life history research. Extrinsic threat from COVID-19 (knowing those hospitalized or dead) was positively associated with ideal number of children. Existential threat (measured via climate change beliefs) was not clearly associated with reproductive decision-making. Taken together, these results provide evidence that reproductive decision-making shifts are functionally attuned to historically recurrent mortality threats like pandemics, but not to more novel, species-wide threats like climate change.

Myers and Emmott explored how new mothers' social communication impacted postnatal depressive symptoms during London's first pandemic lockdown. The authors acknowledged that while humans are cooperative childrearers, pandemic mandates have severely limited in-person contact. Seeing more social network-members in person or communicating more with those not visited with was linked to fewer depressive symptoms in new mothers. However, contact with a greater proportion of relatives was positively associated with depressive symptoms, suggesting that kin may have sought to visit particularly those mothers who needed it the most. Rich qualitative data in Myers and Emmott's article also illustrated themes in COVID-19 lockdown experiences. For example, participants wrote about benefits of the lockdown, like increased time to bond with their baby. They also illustrated lockdown burdens, like the obligation to “constantly mother,” inadequate social support, and missed developmental opportunities for their children. A substantial number of women in the sample met diagnostic criteria for postnatal depression, reflecting a rise in rates seen in other samples collected during the pandemic. Taken together, these results suggest that lockdown has negatively affected mothers' well-being and that peer network members' support is needed to help buffer these impacts.

Not everything is necessarily worse during pandemics. Freitas et al. conducted a longitudinal study before and during the pandemic focusing on anxiety, premenstrual symptoms, and eating behavior in young Brazilian women. They found that anxiety/stress, uncontrolled and emotional eating, and desire for sweet and fatty food were higher before the pandemic. The traditional food, social interaction, and support of living back together in one's family home might buffer people from the stresses of the pandemic, particularly in small-city contexts.

## Conclusion

This Research Topic has gone a long way into offering new high-quality theoretical insights and empirical findings stemming from an integrative evolutionary approach that can contribute to the way psychological and behavioral sciences predict, model, and deal with the current and future pandemics. Forty-three authors contributed to this Research Topic, reporting findings from Australia, Brazil, Croatia, Poland, Serbia, the UK, and the US. We thank the authors, reviewers, and external editors who accepted the challenge of approaching the current COVID-19 pandemic from an evolutionary perspective. It was not an easy task because there is limited existing evolutionary research on pandemics, but this article collection provides examples of the many ways in which evolutionary principles can help advance psychological and behavioral science applied in a pandemic context.

## Author Contributions

All authors contributed to writing this article.

## Conflict of Interest

The authors declare that the research was conducted in the absence of any commercial or financial relationships that could be construed as a potential conflict of interest.

